# Lifestyle and Genetic Factors Modify Parent-of-Origin Effects on the Human Methylome

**DOI:** 10.1016/j.ebiom.2021.103730

**Published:** 2021-12-06

**Authors:** Yanni Zeng, Carmen Amador, Chenhao Gao, Rosie M. Walker, Stewart W. Morris, Archie Campbell, Azra Frkatović, Rebecca A Madden, Mark J. Adams, Shuai He, Andrew D. Bretherick, Caroline Hayward, David J. Porteous, James F. Wilson, Kathryn L. Evans, Andrew M. McIntosh, Pau Navarro, Chris S. Haley

**Affiliations:** a Faculty of Forensic Medicine, Zhongshan School of Medicine, Sun Yat-Sen University, Guangzhou 510080, China; b Guangdong Province Translational Forensic Medicine Engineering Technology Research Center, Zhongshan School of Medicine, Sun Yat-Sen University, Guangzhou 510080, China; c Guangdong Province Key Laboratory of Brain Function and Disease, Zhongshan School of Medicine, Sun Yat-Sen University, Guangzhou 510080, China; d MRC Human Genetics Unit, Institute of Genetics and Cancer, University of Edinburgh, Edinburgh, UK; e Centre for Genomic and Experimental Medicine, Institute of Genetics and Cancer, University of Edinburgh, Edinburgh, UK; f Centre for Clinical Brain Sciences, Chancellor's Building, The University of Edinburgh, Edinburgh, UK; g Genos Glycoscience Research Laboratory, Borongajska cesta 83h, 10000 Zagreb, Croatia; h Division of Psychiatry, University of Edinburgh, Edinburgh, United Kingdom; i Sun Yat-sen University Cancer Center, State Key Laboratory of Oncology in South China, Collaborative Innovation Center for Cancer Medicine, Guangdong Key Laboratory of Nasopharyngeal Carcinoma Diagnosis and Therapy, Guangzhou, 510060, China; j Centre for Global Health Research, Usher Institute, University of Edinburgh, Edinburgh, UK; k Roslin Institute and Royal (Dick) School of Veterinary Studies, University of Edinburgh, Edinburgh, UK

**Keywords:** parent-of-origin effect, DNA methylation, interaction (modification) effect, mQTL, DNA methylation machinery genes, smoking

## Abstract

**Background:**

parent-of-origin effects (POE) play important roles in complex disease and thus understanding their regulation and associated molecular and phenotypic variation are warranted. Previous studies mainly focused on the detection of genomic regions or phenotypes regulated by POE. Understanding whether POE may be modified by environmental or genetic exposures is important for understanding of the source of POE-associated variation, but only a few case studies addressing modifiable POE exist.

**Methods:**

in order to understand this high order of POE regulation, we screened 101 genetic and environmental factors such as ‘predicted mRNA expression levels’ of DNA methylation/imprinting machinery genes and environmental exposures. POE-mQTL-modifier interaction models were proposed to test the potential of these factors to modify POE at DNA methylation using data from Generation Scotland: The Scottish Family Health Study(N=2315).

**Findings:**

a set of vulnerable/modifiable POE-CpGs were identified (modifiable-POE-regulated CpGs, N=3). Four factors, ‘lifetime smoking status’ and ‘predicted mRNA expression levels’ of *TET2, SIRT1* and *KDM1A*, were found to significantly modify the POE on the three CpGs in both discovery and replication datasets. We further identified plasma protein and health-related phenotypes associated with the methylation level of one of the identified CpGs.

**Interpretation:**

the modifiable POE identified here revealed an important yet indirect path through which genetic background and environmental exposures introduce their effect on DNA methylation, motivating future comprehensive evaluation of the role of these modifiers in complex diseases.

**Funding:**

NSFC (81971270),H2020-MSCA-ITN(721815), Wellcome (204979/Z/16/Z,104036/Z/14/Z), MRC (MC_UU_00007/10, MC_PC_U127592696), CSO (CZD/16/6,CZB/4/276, CZB/4/710), SFC (HR03006), EUROSPAN (LSHG-CT-2006-018947), BBSRC (BBS/E/D/30002276), SYSU, Arthritis Research UK, NHLBI, NIH**.**

**Research in context**

**Evidence before this study**

Previous population studies showed that parent-origin-effects (POE) on human methylome can be widespread and affect health-related traits and diseases. Whether the POE remain stable throughout the life or can be modified by genetic or environmental factors was largely unknown. The POE are mainly introduced by imprinting. A case study reported one imprinted locus where the imprinting status was modified by genetic variants and environmental factors such as maternal nutrition and maternal age, and that the modulated imprinting status influenced childhood BMI. Whether the POE at DNA methylation levels could be modified and which genetic/environmental factor could introduce this POE-specific modification effect remained unknown.

**Added value of this study**

By systematically screening 101 genetic and environmental factors in a large cohort(GS:SFHS) we provided population-level replicated evidence that those measuring lifestyle (smoking) and predicted expression of DNA methylation- or imprinting- machinery genes are amongst the factors that can modulate the POE of mQTLs for a set of CpG sites. We found those modifiable-POE-regulated CpGs are also phenotypically relevant –one is associated with the plasma levels of CLEC4C and health-related phenotypes such as HDL levels.

**Implications of all the available evidence**

The modifiable POE identified here revealed an important yet indirect path through which genetic background and environmental exposures introduce their effect on DNA methylation and their associated phenotypes. This also provided a paradigm for further studies to explore how environmental and genetic effects can be integrated at methylation level.

Alt-text: Unlabelled box

## 1 Introduction

**Figure 1:**
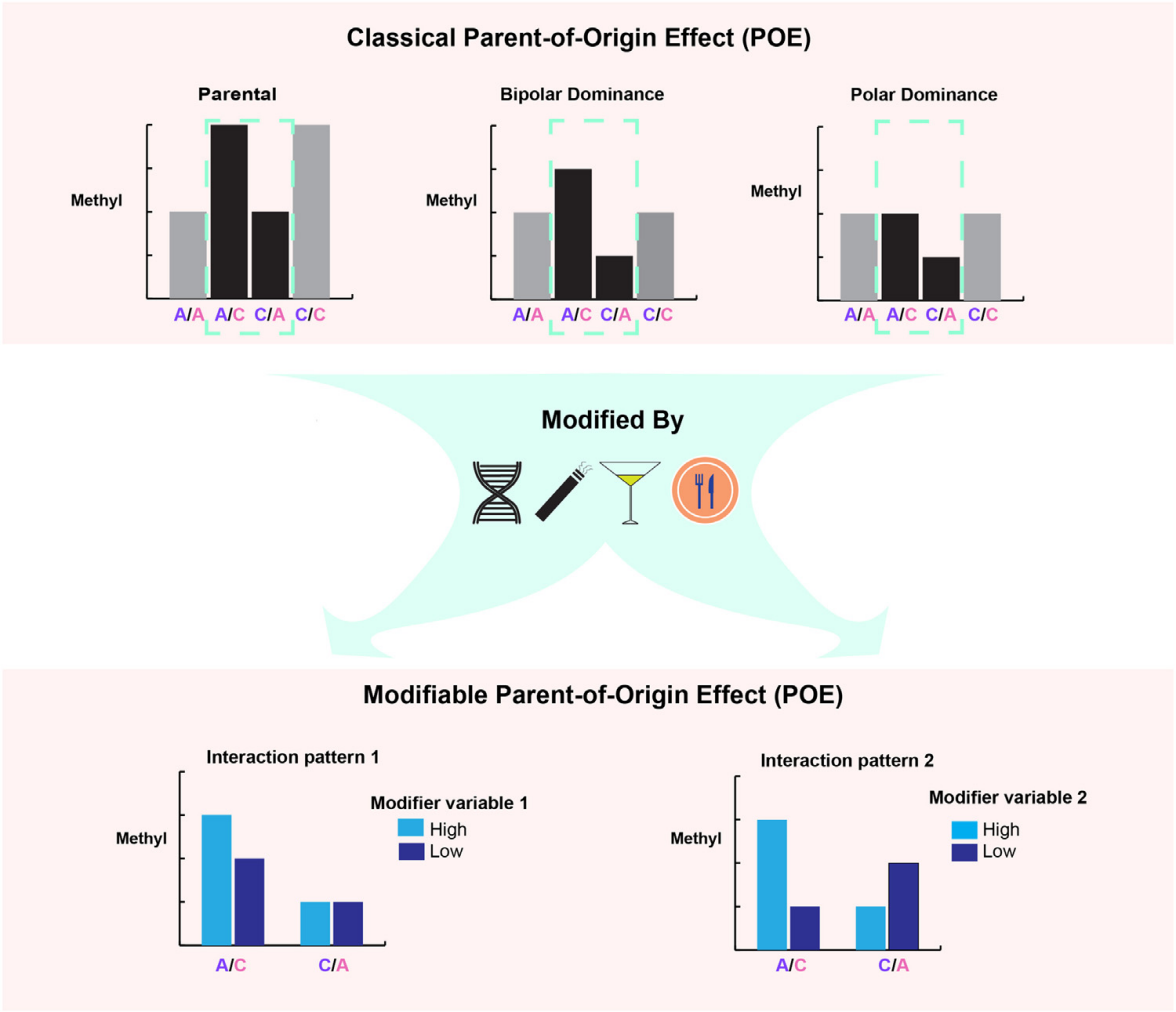
Patterns of classical and modifiable parent-of-origin effect (POE) regulation on DNA methylation. X axis: mQTL genotypes, left purple: paternal allele, right pink: maternal allele. Y axis: methylation level of the regulated CpG. Upper panel: classical POE patterns including parental and complex (dominance) POE patterns. Parental patterns show two levels of methylation depending on the expressed allele and the allelic effect. Complex POE manifests as the two homozygous groups having the same methylation level whereas the heterozygous groups are different. Dashed box: difference between methylation level of heterozygous groups of the mQTL is the hallmark of POE. Bottom: scenarios when the POE is modified by genetic or environmental factors, leading to the alteration of POE for different levels of the modifier.

Illustrating the sources of variation in DNA methylation lays the foundation for understanding epigenetic-based biomarkers for disease risk and progress prediction [1,2]. DNA methylation is known to be influenced by additive and non-additive genetic and environmental factors [3], [4], [5]. As a special form of non-additive genetic effects, parent-of-origin effects (POE) on the human methylome manifest as differences in methylation levels between the reciprocal heterozygotes of the mQTL depending on the allelic parent-of-origin (Figure 1) [6]. Through selectively silencing the maternal or paternal allele, genomic imprinting has been considered as the major driving force creating the POE phenomenon [6]. We and others have shown that POE-influenced methylation sites are not rare, many are regulated by mQTLs, and that they follow one of the three classical imprinting patterns: parental, bipolar dominance and polar dominance (Figure 1) [4,7,8]. Although they only comprise a small proportion of the genome, POE (imprinting)-regulated CpGs and genes have been found to be important for developmental, metabolic and behavioral traits [4,9].

Despite their functional importance, the POE patterns in those POE (imprinting)-influenced regions can fluctuate as a consequence of genetic and environmental variation. Previous studies have reported that a large fraction of imprinted regions deviated from mono-allelic expression and that birth phenotypes were associated with the extent of this deviation [10]. A case study on an imprinting influenced long non-coding RNA, lncRNA nc886, found that the imprinting status of this locus is tunable by both genetic variants and environmental factors such as maternal nutrition and maternal age[11]. Importantly, the altered POE has phenotypic consequences: loss of imprinting of nc886 in infants at birth resulted in increased body mass index (BMI) in childhood [11]. For the majority of other POE-influenced regions, however, whether POE are modifiable under certain conditions remains unknown. Here we aim to explore modifiable POE, manifesting as the altered methylation difference between reciprocal heterozygotes of the mQTL due to effects from certain genetic or environmental modifiers (Figure 1), which potentially represents an important layer of POE-related regulation requiring systematic examination.

To search for modifiable POE on CpGs, a key question is which modifiers may have the potential to regulate the POE. Genomic imprinting, which likely underlies the POE, involves complex and multi-stage DNA methylation reprogramming processes, from the slow erasure of methylation at primordial germ cell stage, to the establishment of imprinted methylation signatures at germ cell stage, followed by pre-implantation maintenance of the imprinted methylation pattern during the global demethylation event, which is subsequently maintained post-implantation [12]. A number of gametic and zygotic genetic factors were found to be involved in these processes, such as those functioning in folate metabolism, the DNA methylation machinery (writers, erasers) and the proteins with which they interact [12]. Additionally, imprinting-related processes have also been found to be sensitive to environmental insults, such as the stress induced by assisted reproductive technologies, nutritional deficiency and adverse exposures [12], [13], [14]. Given that previous studies of modifiers of genomic imprinting were mostly case studies of individual factors, a systematic and population-wide scanning for genetic and environmental modifiers of POE is essential to fully characterize POE regulation.

**Figure 2:**
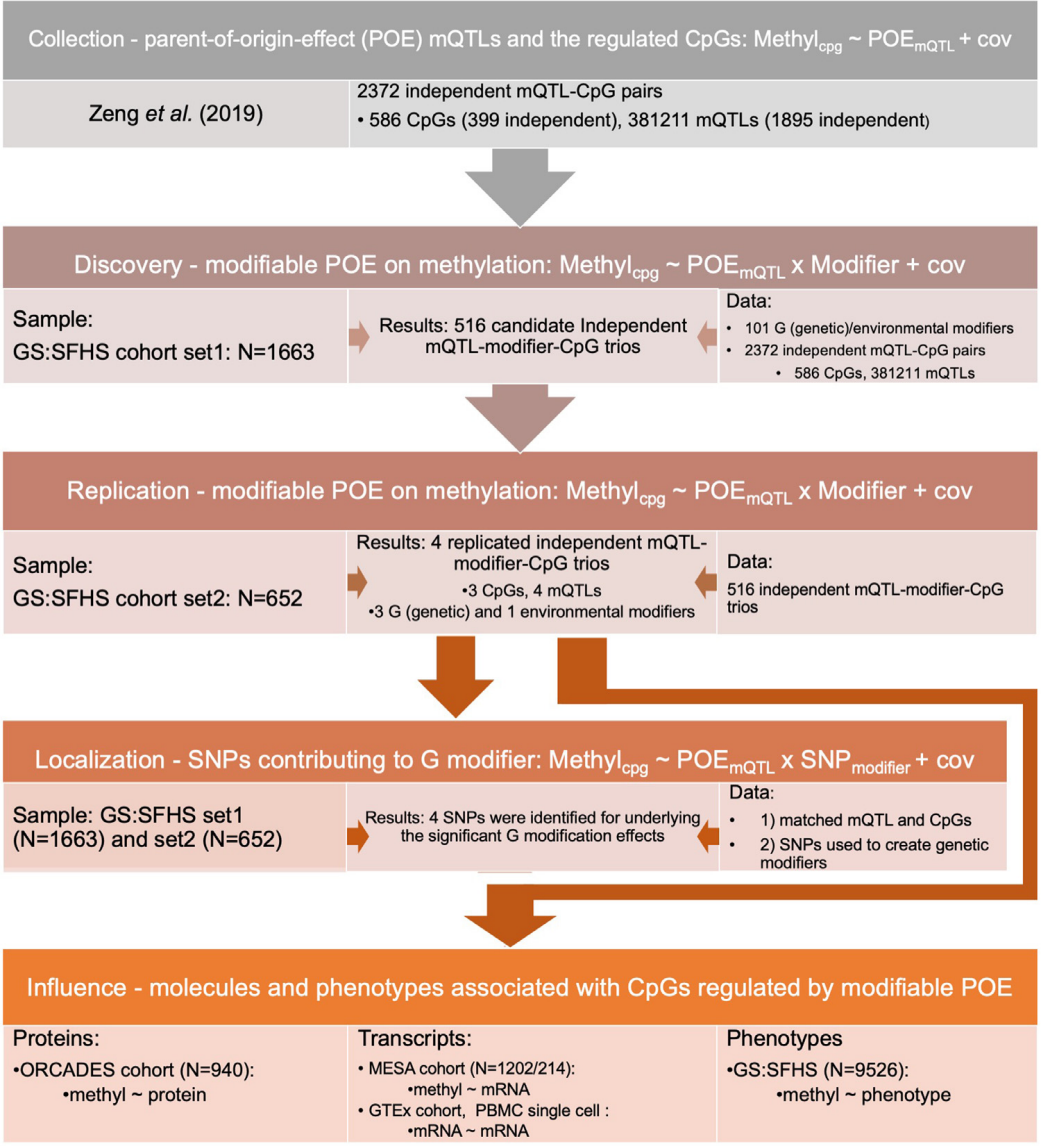
Design of the study. Cov: covariates fitted in the model. Zeng *et al.*(2019): the study which reported CpGs regulated by POE and the mQTLs that induce the POE for 586 CpGs (reference 4).

In this study, we used Generation Scotland: The Scottish Family Health Study (GS:SFHS), a large family-based population cohort with extensive environmental and phenotypic records [15,16], genome-wide genotypes and DNA methylation data [17,18], to identify genetic and environmental factors that modify the POE on the human methylome (Figure 1). Figure 2 illustrates the study design. Based on the 2372 previously identified independent mQTL-CpGs pairs containing mQTLs with parent-of-origin effect (1895 independent mQTLs; 381211 SNPs in total) and their regulated CpGs (399 independent CpGs; 586 CpGs in total) [4], we proposed an interaction model which tests for significant interaction effects (Figure 1) between each of the 101 candidate environmental/genetic modifiers available in GS:SFHS and the parent-of-origin effect of each mQTL on the corresponding targeted CpG. Significant results from discovery samples (GS:SFHS set1, N=1663) were tested in replication samples (GS:SFHS set2, N=652). After modifiable-POE-regulated CpGs were identified, we further characterized those CpGs by examining their associations with potentially important plasma proteins measured by Olink panels (designed to capture proteins relevant to diseases and important biological processes) and associations with other health-related phenotypes to evaluate the phenotypic relevance for this special class of CpGs.

## 2 Methods

### 2.1 Ethics

GS:SFHS study was ethically approved by the Tayside Research Ethics Committee (reference 05/S1401/89). Participants all gave written consent after having an opportunity to discuss the research and before any data or samples were collected. ORCADES study received ethical approval from the appropriate research ethics committees in 2004. Informed consent was provided by all participants.

### 2.2 Population samples

GS:SFHS is a deeply phenotyped family-based population cohort with genome-wide genotypes available for 19994 participants[15,16]. Participants were recruited from the registers of collaborating general practices in Scotland between 2006 and 2011. At least one first-degree relative aged 18 or over was required to be identified for each participant [15,16]. Among GS:SFHS participants, 9526/19994 have DNA methylation data in blood available(see below), 7106/19994 have the parent-of-origin allelic inheritance of all imputed common SNPs successfully inferred with high accuracy [4,15,17,18]. This lead to a total of 2315 GS:SFHS participants with both DNA methylation and parent-of-origin phased genotypes available, which allowed analyses to identify CpGs regulated by modifiable POE here. We categorized those 2315 participants into two groups, according to their collection batches for DNA methylation data. We used those from the first batch as the discovery dataset(N=1663), and those from the second batch as the replication dataset(N=652). We additionally make full use of all 9526 participants with methylation data and the collection of phenotype information available in GS:SFHS to explore health-related phenotypes associated with modifiable-POE-regulated CpGs.

ORCADES is a family-based cross-sectional study which recruited 2078 participants between 2005 and 2011 from the Orkney Isles in northern Scotland [19]. Proteomic and DNA methylation data were available for a subset of 940 participants and were used here for association test between modifiable-POE-regulated CpGs and plasma proteins.

#### 2.2.1 GS:SFHS cohort: genotypes and inference of parent-of-origin transmission of alleles in offspring

Genome-wide genotypes were generated using the Illumina Human OmniExpressExome -8- v1.0 array [20]. Phasing, imputation and quality control were described previously [4,21]. In total, 7108491 high-quality imputed common SNPs (MAF ≥.01, info score ≥ 0.8) for 19994 participants were available for subsequent analyses. Among those individuals, there were 7139 offspring with at least one of their parents genotyped in GS:SFHS, which allowed us to successfully infer parent-of-origin allelic inheritance of all imputed common SNPs in 7106 offspring with high accuracy [4].

#### 2.2.2 GS:SFHS cohort: DNA methylation

In GS:SFHS, genome-wide DNA methylation data were produced through a related Stratifying Resilience and Depression Longitudinally (STRADL) project [18]. In 2016, the first batch of methylation data was generated on 5081 participants. These were used as discovery subset. 1663 of these participants also had imputed genotype information with parent-of-origin alleles successfully inferred and were used for the scanning for modifiable POE here. In 2019, another batch of methylation data was generated on an independent subset of 4445 participants. These data were used as replication subset. 652 out of these 4445 participants had imputed genotype information with parent-of-origin alleles successfully inferred. Based on a pipeline proposed previously [4], the two datasets were generated, processed and quality controlled in consistent way [22], which was briefly described in Text s1.

#### 2.2.3 GS:SFHS cohort: Environmental/genetic modification variables Environmental modifier variables

Environments and life events can be important sources of modifiers for POE [12], [13], [14]. The core GS:SFHS cohort has rich collections of environmental variables [15]. Moreover, 98% of GS:SFHS participants gave informed consent for data linkage with historic Scottish birth cohorts which contain collections of birth and maternity information(Text s2). In total, we were able to collect 75 environmental variables and used them in downstream analyses. The variables ranged from baseline variables (sex, age etc.), medication (blood pressure lowering medication, antidepressants etc,), lifestyle (smoking, exposure to smoking, alcohol consumption, diet, physical activity etc.), living/socio-economic status (number of people sharing the house, job type, parents’ health conditions etc.), birth-related phenotypes (birth weight, gestational age, etc.) and menarche/menopause-related events. A full list of environmental variables is given in Table s1.

#### 2.2.4 Genetic modifier variables

Genetic factors that are involved in establishment and maintenance of imprinting can be important sources of modifiers for POE [12]. We considered two categories of genetic modifiers:

1) Predicted mRNA expression levels of 17 DNA methylation or imprinting-specific machinery genes. DNA methylation or imprinting-specific machinery genes play crucial roles in imprinting [12]. Since GS:SFHS participants don't have directly measured mRNA expression levels and prediction model has been proven to be effective in evaluating genetically-determined expression variation [23], PrediXcan was applied to impute gene expression levels of machinery genes in blood [23]. A list of 30 genes was collated from the literature on the basis of having critical roles in DNA methylation in general, such as methylation writers and erasers, or in imprinting specifically (Table s2). Among them 17 have their mRNA expression levels in blood successfully predicted(details in Text s2 and Table s1).
2) Nine genetic risk scores (PRSs) for folate metabolism. Folate and one-carbon metabolism play important roles in DNA methylation turnover and genomic imprinting [12]. Genetic variation can contribute to individual variation in this pathway. PRSs were therefore created to represent a participant's genetic predisposition for functions relating to folate metabolism (details in Text s2 and Table s1.).

### 2.3 Statistics

#### 2.3.1 POE-mQTL-modifier interaction models

We applied a POE-mQTL-interaction model to test whether environmental or genetic factors could modify the POE induced by mQTL on CpGs. The model built on a linear regression model that we used to identify POE-specific mQTL-CpG pairs (mQTL with a parent-of-origin effect and the CpG it regulated) in our previous study [4,24]:

*model 1 - non-interaction model:*

$$Methyl_{cpg}=Add_{mQTL}+Dom_{mQTL}+POE_{mQTL}+covariates+error$$

Where for the additive genetic variable (Add_mQTL_), the dominance genetic variable (Dom_mQTL_) and the parent-of-origin effect (POE_mQTL_), genotypes were coded as below:

**Table:** 

	AA	Aa	aA	aa
Additive	0	1	1	2
Dominance	0	1	1	0
Parent-of-origin	0	-1	1	0

In this study, we applied an interaction model that additionally includes a modifier variable, *Mod*, and its interaction with the additive genetic effect and the POE:

*model2 - interaction model:*

$$\begin{array}{c} Methyl_{cpg}=Add_{mQTL}+Dom_{mQTL}+POE_{mQTL}+Mod+Add_{mQTL}xMod\\ +POE_{mQTL}xMod+covariates+error \end{array}$$

Where the modifier variable (*Mod*) was one of the environmental/genetic variables collected/derived as described in the section ‘GS:SFHS cohort: Environmental/genetic modification variables’. Covariates included age, sex, cell proportions, smoking variables (‘pack years’ and ‘lifetime smoking status’, which were not included as covariates when they were the tested modifier factor) and principal components (PCs) derived from an OMIC-relationship-matrix (ORM) created in OSCA [25] using all measured DNA methylation sites. To avoid removing genetic signals of interest by fitting ORM-PCs, we only included ORM-PCs among the top 20 ORM-PCs that were not significantly associated with any common SNP(determined through performing GWAS for ORM-PCs). We applied this model and tested the significance of the interaction effect between each mQTL's POE and the modifier variable (*POE_mQTL_ x Mod,* short for *POE_mQTL_ x Modifier*) on the corresponding CpG. Throughout the paper, we described an interaction effect (*POE_mQTL_ x Mod*) on a CpG as a **mQTL-modifier-CpG trio**. The mQTL-CpG pairs tested were the 2372 independent POE-specific mQTL-CpG pairs which we reported previously [4]. The interaction between the additive effect and the modifier (*Add_mQTL_ x Mod*) was jointly fitted in the model. For simplicity we did not fit an interaction between the dominance effect and the modifier (*Dom_mQTL_ x Mod*) in the global scan, but we did this in sensitivity tests for the significant mQTL-modifier-CpG trios we identified. The results indicated only a minor contribution of the *Dom_mQTL_ x Mod* effect (Table s3). Multiple testing correction was performed by a combination of a global permutation test and mQTL-modifier-CpG trio-specific permutation tests for *POE_mQTL_ x Modifier* interaction effect at discovery stage(FDR≤0.05, details see Text s3, Table s4). A successful replication required to both reach statistical significance (FDR≤0.05) and patten consistency (details in Text s3, Table s4-6). Visualization of the results was performed using the R package coMET and ggplot2 [26,27].

#### 2.3.2 Identification of proteins and mRNAs associated with CpGs regulated by modifiable POE *ORCADES cohort- DNA methylation and proteomic data:*

DNA methylation levels was measured from whole blood samples using Illumina MethylationEPIC Array for 794627 CpG sites in 1052 samples (quality control and pre-correction in Text s4). Abundance of plasma proteins was measured from the fasted EDTA plasma samples for a subset of 1051 participants using Olink Proseek Multiplex cardiometabolic, cell regulation, cardiovascular 2 and 3, developmental, immune response, inflammation 1, metabolism, neuro exploratory, neurology, oncology and organ damage panels. The raw NPX values were used in analysis.

#### 2.3.3 Association between DNA methylation and plasma protein levels

A linear mixed model was used to compute methylation residuals after adjusting for genetic structure in ORCADES by fitting a random effect represented in the genomic relationship matrix. To assess whether the CpGs (N_cpg_=3) significantly regulated by interaction effects (*POE_mQTL_ x Mod*) were associated with the abundance of any plasma protein (N_protein_=1092), the association between adjusted methylation of CpGs of interest with each measured protein was tested using a linear regression model in 940 participants where DNA methylation and proteomic data were simultaneously available:

Methyl=protein+cellproportion+age+sex+seasonofsampling+smokingstatus+error

The Bonferroni method was applied to correct for multiple testing (N_correction_=3 × 1092=3276).

#### 2.3.4 Correlations between DNA methylation vs mRNA levels

Transcriptomic and DNA methylation data from human peripheral monocytes and T cells in the Multi-Ethnic Study of Atherosclerosis (MESA) study were downloaded from the NCBI GEO database (Series GSE56047 and GSE56580) [28]. mRNA was measured using the Illumina HumanHT-12 v4 Expression BeadChip, DNA methylation levels were measured using the Illumina HumanMethylation450 BeadChip [28]. Quantile-normalized signal for mRNA (log2 transformed) and DNA methylation data (M-values) were simultaneously available for peripheral monocytes (CD14+) in 1202 participants and for peripheral T cells (CD4+) in 214 participants and were used to calculate the Spearman correlation between DNA methylation and mRNA.

#### 2.3.5 Correlations between mRNAs levels

At the population-level, correlations between mRNA levels of target genes were calculated using the Spearman method on GTEx whole-blood data using the GEPIA portal [29]. At single cell level, a normalized single-cell matrix for 63628 peripheral blood mononuclear cells (PBMC) from a healthy donor were obtained from the website http://tisch.comp-genomics.org/gallery/. Feature counts for each cell were normalized by ‘LogNormalize’, a global-scaling method that normalizes the cellular feature expression by dividing the total counts for that cell using R package Seurat (https://satijalab.org/seurat/), multiplied that by a scale factor (10000 by default), followed by a natural-log transformation. Spearman's correlation between mRNA levels of target genes was calculated using cells where normalized expression levels of both genes was larger than zero.

#### 2.3.6 Phenome-wide association test for DNA methylation sites regulated by modifiable POE

We collected 79 phenotypes measured in GS:SFHS (recorded dataset and linked health records) to identify phenotypes correlated with DNA methylation levels at CpG sites targeted by modifiable POE. The full list of phenotypes tested can be found in Table s7. The correlation was tested by regressing the adjusted M-values of methylation sites on each phenotype variable. Covariates included age, age^2^, cell proportion, sex, top 20 ORM-PCs and smoking variables (‘pack years’ and ‘lifetime smoking status’; these were not included as covariates when they were the tested phenotype). The test was performed in the discovery and replication samples separately and meta-analyzed using a random effect model using the R package metafor [30]. The sample size varied depending on the number of missing samples for each specific phenotype (Table s8). The Bonferroni method was used for multiple testing correction (N_correction_=79*3=237).

### 2.4 Access to data

Summary statistics supporting the conclusions of this article are included within the article and its additional files. GS:SFHS: the Generation Scotland data used here are available from the MRC IGC Institutional Data Access / Ethics Committee for researchers who meet the criteria for access to confidential data. Applications should be submitted to the Generation Scotland Access Committee (access@generationscotland.org). The managed access process ensures that approval is granted only to research which comes under the terms of participant consent which does not allow making participant information publicly available. ORCARDES: there is neither research ethics committee approval, nor consent from individual participants, to permit open release of the individual level research data underlying this study. Please contact the QTL Data Access Committee (accessQTL@ed.ac.uk) for further information if required.

### 2.5 Role of funders

The Funders had no role in the study design; the collection, the analysis, the interpretation of the data, and the writing of the paper.

## 3 Results

### 3.1 DNA methylation sites targeted by modifiable POE

In GS:SFHS, 2315 participants have both parent-of-origin phased genotypes and DNA methylation data available. DNA methylation levels were obtained in two batches, for 1663 participants in the first batch in 2016 and for 652 in the second batch in 2019. These were used as the discovery and the replication datasets respectively. Participants in the discovery dataset were genetically independent from participants in the replication dataset (relatedness < 0.05). The two datasets have similar distribution for age: 34.5(18-56) in discovery set vs 34.2(18-58) in replication set (P=0.61, t-test) and gender: female:male: 62%: 38% in discovery set and 58%: 42% in replication set (P=0.08, Chi-squared test).

We utilized information on 75 environmental and 26 genetic variables to test if any of them significantly modified the parent-of-origin effects of the mQTLs on methylation level of the targeted CpGs, altering the methylation difference between reciprocal heterozygotes of the mQTLs (Figure 1). The environmental candidate modifiers reflected the environments/events the participants have been exposed to or have experienced, including those measuring baseline non-genetic effects (sex, age), medication, lifestyle, socioeconomic status, birth-related phenotypes (measured before or after participants’ birth) and menarche/menopause-related events; the genetic candidate modifiers were genetic factors known to be involved in DNA methylation and imprinting processes, including ‘predicted whole blood mRNA expression levels’ of DNA methylation- or imprinting-specific machinery genes, and PRSs for folate-related metabolism (details in Methods and Table s1).

POE-mQTL-modifier models were applied to identify modifiable POE on DNA methylation, with age, sex, cell proportions, smoking variables (‘pack years’ and ‘lifetime smoking status’, which were not included as covariates when they were the tested modifier factor) and ORM PCs (see Methods) fitted as covariates. At the discovery stage, a global permutation test was combined with mQTL-modifier-CpG trio-specific permutation tests (Text s3) to determine mQTL-modifier-CpG trios for significant *POE_mQTL_ x Modifier* interaction effect on DNA methylation (see Methods). At the replication stage, a successful replication required both statistical significance and pattern consistency (direction-of-effect) for both the main POE and the *POE_mQTL_ x Modifier* interaction effect in tested mQTL-modifier-CpG trios.

**Figure 3:**
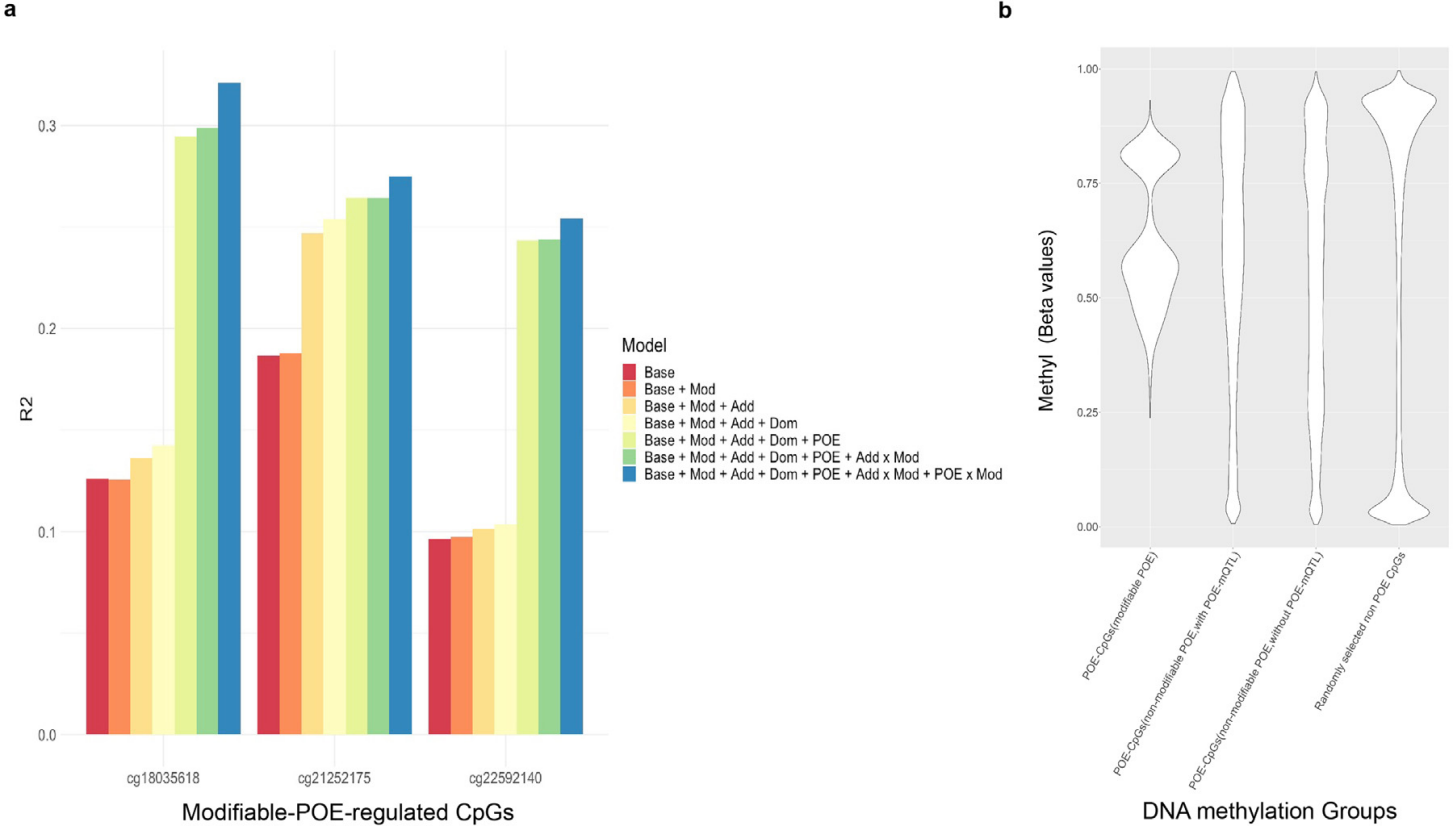
CpGs regulated by the modifiable-POE. a. Proportion of methylation variation explained by different models for the three CpGs regulated by modifiable-POE. The ‘Base model’ accounted for age, sex, cell proportion, smoking variables (‘pack years’ and ‘lifetime smoking status’. These were not included as covariates when they were the tested modifier factor) and principal components of the OMIC (DNA methylation)-relationship-matrix. Mod: modifier; Add: additive genetic effect, Dom: dominance genetic effect. Add x Mod: interaction between additive genetic effect and the modifier; POE x Mod: interaction between parent-of-origin genetic effect and the modifier. b. Distributions of methylation levels of CpGs regulated by modifiable POE (N_CpG_=3), CpGs regulated by POE from known mQTLs but the POE is not modifiable (N_CpG_=583), CpGs regulated by POE but without an mQTL identified (the CpGs were reported in ref 4: Zeng *et al.* (2019), N_CpG_=398) and randomly selected non-POE CpGs from all QCed array probes (N_CpG_=10,000). Unrelated individuals from the GS:SFHS discovery subset were used to produce the plot.

In total, four mQTL-modifier-CpG trios reached significance in the discovery sample (*P_global(FDR_adjusted)_ < 0.05, P_trio-specific(Bonferroni_adjusted)_ < 0.05*, permutation) and were successfully validated in the replication sample (Table 1, Text s2, Tables s4-6). Sensitivity analysis removing ORM PCs from the model obtained consistent results (Table s9). These four mQTL-modifier-CpG trios involved one environmental modifier: ‘lifetime smoking status’; and three genetic modifiers: ‘predicted mRNA expression levels’ of *SIRT1* (a gene that protects methylation at imprinted loci by directly regulating acetylation of DNMT3L [12,31]), *TET2* (a DNA demethylation gene [12,32]) and *KDM1A* (a gene involved in removal of methylation and histone H3K4 in imprinted genes[12,33]). The three CpGs, cg18035618, cg21252175, and cg22592140 were the methylation sites affected by these modification effects (Table 1), with the *POE_mQTL_ x Modifier* interaction effect explaining between 1.0% to 2.2% of their methylation variation (Figure 3a, Table s3). These CpGs displayed intermediate methylation levels, when compared with the less-peaked distribution of methylation levels of POE-regulated CpGs not influenced by modifiable effects, or the bimodally distributed methylation level of genomic CpGs not influenced by POE (Figure 3b). Genomic annotation of the three CpGs was displayed in Figure s1 and Table s10. Two of the three CpGs (cg18035618 and cg22592140) were located within parent-of-origin differentially methylated regions [34] and imprinting control region (ICR) of imprinted genes *GNAS* and *MEST* [*35*]*,* within 500bp distance of the Fantom5 CAGE peaks for promoters [36], and overlapped with H3k4me1 and H3k4me3 histone modification regions [37]. The third CpG (cg21252175) was located within CCCTC-binding factor (CTCF) binding site, H3k4me and H3k27ac histone modification regions [37].

**Table 1:** Genetic/environmental modifiers displaying a significant interaction with the POE(mQTL) and the CpGs the interaction effects affect.

mQTL-CpG-modifer trio information								Discovery					Replication			
CpG: id	CpG-chr	CpG-POS	mQTL:id	mQTL- chr	mQTL- POS	Modifier	Modifier type	P(dis,t-test)	Est(dis)	N(dis)	P(trio-specific, permutation)	Adjusted P(trio-specific. permutation)	P(rep)	Est(rep)	N(rep)	Adjusted P(rep, t-test)
cg22592140	7	130132419	rs142053217	7	130258926	mRNA expression of KDM1A	Genetic	1.35E-06	6.35	1607	1.00E-05	1.09E-02	6.09E-04	6.52	645	2.79E-02
cg21252175	15	64973903	rs111834387	15	64062211	mRNA expression of TET2	Genetic	1.54E-06	3.77	1607	<1e-5	< 0.01	7.76E-11	13.80	647	2.43E-08

cg18035618	20	57415978	rs117020124	20	57347435	Lifetime smoking status	Env	9.94E-07	-0.04	1607	<1e-5	< 0.01	1.42E-03	-0.05	647	4.85E-02
			rs117823351	20	57369773	mRNA expression of SIRT1	Genetic	3.68E-10	-0.24	1607	<1e-5	< 0.01	1.01E-03	-0.22	646	3.95E-02

CpG-POS/mQTL-POS: genomic position of the CpG and mQTL(hg19). Env: environmental modifier. dis: discovery sample, rep: replication sample. N: sample size. P(dis, t-test)/P(rep, t-test) and Est(dis)/Est(rep): P values (t test) and coefficients for the *POE_mQTL_ x Modifier* effect in the interaction model in discovery/replication samples. P(trio-specific, permutation): P value for the *POE_mQTL_ x Modifier* effect from the mQTL-CpG-modifer trio-specific permutation test. Adjusted P (trio-specific, permutation): adjusted P(trio-specific, permutation) using Bonferroni method(Text s3). Adjusted P(rep, t-test): adjusted P(rep, t-test) for interaction effect at replication dataset using the FDR method. Genomic position of the significant genetic modifiers: *KDM1A*: chr1: 23345941-23410182; *TET2*: chr4: 106067,032-106200973; *SIRT1*: chr10: 69644427-69678147.

To test 1) whether the three CpGs identified represent hotspot DNA methylation regions for being regulated by the modifiable POE (hopspot CpGs: CpGs with higher chance to be targeted by modifiable POE than other CpGs), and 2) whether the four modifiers identified represent hotspot factors to introduce the modification effect (hotspot factors: modifiers with higher chance to modify POE than other potential modifiers), we ranked all mQTL-modifier-CpG trios including those not reaching the significance threshold by P values of the *POE_mQTL_ x Mod* interaction. We found that the *POE_mQTL_ x Mod* interactions targeting the three modifiable-POE-regulated CpGs ranked significantly higher than interactions targeting POE-CpGs with known mQTLs but not influenced by modifiable POE (P< 2.2 × 10^−16^, Mann-Whitney U test). On the other hand, among the three significant genetic modifiers, *POE_mQTL_ x Mod* interactions involving two of them, ‘predicted mRNA expression levels’ of *TET2* and *SIRT1*, ranked significantly higher than interactions involving other tested modifiers (*P_TET2_* =1.62 × 10^−12^, *P_SIRT1_* =6.22 × 10^−15^, Mann-Whitney U test). A combined collection of smoking-related modifiers also displayed significant enrichment for *POE_mQTL_ x Mod* interactions although lifetime smoking status itself along didn't display the enrichment (*P_smoking-related_* =2.49 × 10^−9^, *P_lifetime_smoking_statu_*_s_ =1, Mann-Whitney U test). Thus, the three CpGs represented hotspots as the targets of *POE_mQTL_ x Mod* interaction effects, modifiers such as smoking-related modifiers and ‘predicted mRNA expression levels’ of *TET2* and *SIRT1* represented important sources of modification effects with which more modification effects can potentially be detected in better powered future studies.

**Figure 4:**
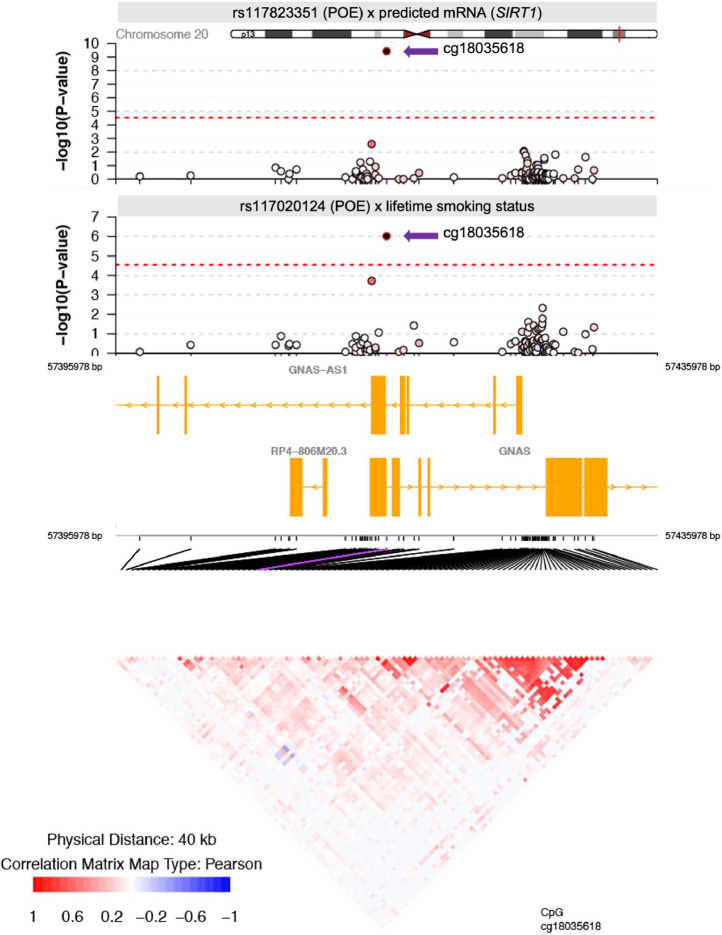
Regional plot of the modifiable-POE affecting cg18035618 and nearby CpGs within a 20kb distance. Top two panels: upper - interaction effect between the POE of cg18035618’s mQTL rs117823351 and ‘predicted mRNA expression levels of *SIRT1’*, bottom - interaction effect between the POE of cg18035618’s mQTL rs117020124 and ‘lifetime smoking status’; –log10 (P-value): minus log10 P-value (t test) of the *POE_mQTL_ x Modifier* interaction effect; Dots show nearby measured CpGs located within a 20kb distance from cg18035618, filling colour represents the correlation of methylation levels with cg18035618: red: positive correlation; blue:negative correlation; white: no significant correlation. Middle panel: genes located within the 40kb genomic region. Bottom panel: correlation matrix between CpGs.

**Figure 5:**
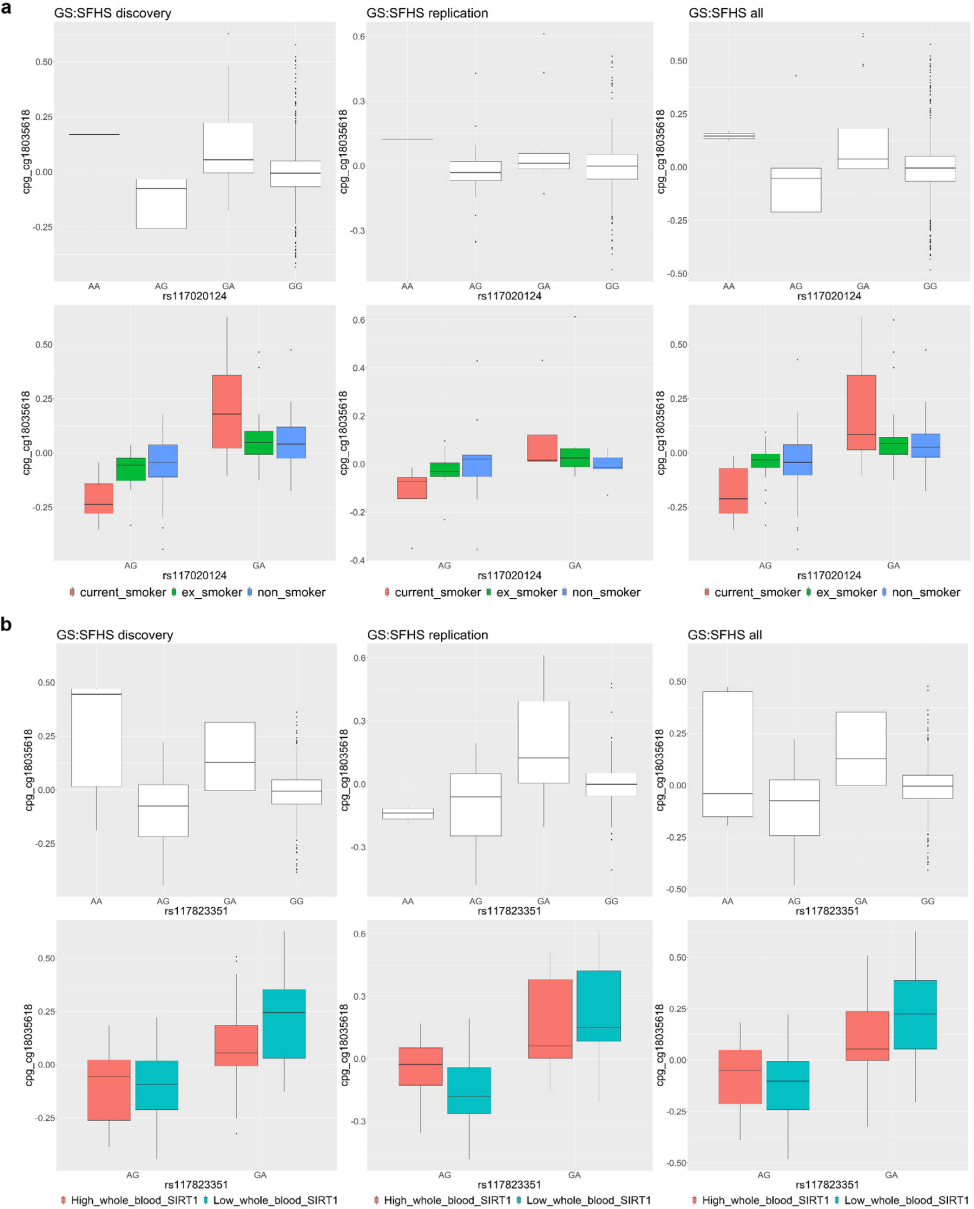
Both environmental and genetic factors significantly modified the POE of mQTLs on cg18036618. a). top:cg18036618 was regulated by the POE of the mQTL rs117020124. bottom: the POE of rs117020124 was modified by lifetime smoking status. The contrast in methylation levels of cg18035618 between heterozygotes of the mQTL rs117020124 is largest in current smoker group. b). top:cg18036618 was regulated by the POE of the mQTL rs117823351. bottom: the POE of rs117823351 was modified by ‘predicted mRNA expression level of *SIRT1*’. The contrast in methylation levels of cg18035618 between heterozygotes of the mQTL rs117823351 is larger in individuals with low SIRT1 expression. High/low expression: participants with predicted expression levels larger than the median were categorized as the high group.

One of the three CpGs, cg18035618, was simultaneously targeted by both environmental and genetic modification effects (Table 1). cg18035618 (hg19: chromosome 20: 57415978) is located in the gene *GNAS* (Figure 4). The methylation level of cg18035618 was significantly modulated by a *POE_mQTL_ x Modifier* interaction between its mQTL, rs117020124, and ‘lifetime smoking status’ (*P_dis_=*9.94 × 10^−7,^ *P_rep_=*1.42 × 10^−3^, t-test, Figure 4, Table 1), with current smokers displaying larger contrast in methylation levels of cg18035618 between heterozygous groups of the mQTL when compared with ex-smokers and never-smokers (Figure 5a). For this CpG a significant *POE_mQTL_ x Modifier* interaction was also detected between another independent mQTL, rs117823351, and ‘predicted mRNA expression levels of *SIRT1*’ (*P_dis_*=3.68 × 10^−10^*, P_rep_=*1.01 × 10^−3^, t-test, Figure 4, Table 1. *SIRT1* is located in chromosome 10), with lower *SIRT1* expression corresponding to an increased contrast of methylation levels between heterozygous groups of the mQTL (Figure 5b). To rule out the possibility that the sharing of the methylation target (cg18035618) by both genetic and environmental modifiers was due to genetically influenced environmental effects (i.e., *SIRT1* expression influences smoking status) [38], we calculated the correlation between ‘lifetime smoking status’ and ‘predicted mRNA expression levels of *SIRT1*’ and found no significant correlation (R=0, *P*=0.968, Pearson).

cg21252175 (hg19: chromosome15: 64973903) is located in the 3’ UTR region of gene *ZNF609* (Figure s2a). For this CpG, a significant *POE_mQTL_ x Modifier* interaction was detected between the mQTL rs111834387 and ‘predicted mRNA expression levels of *TET2*’ (*P_dis_=*1.54 × 10^−6^*, P_rep_*=7.76 × 10^−11^, t-test. *TET2* is located in chromosome 4), with lower expression of *TET2* reducing the contrast in methylation levels of cg21252175 between heterozygous groups of the mQTL (Table 1, Figure s3a). cg22592140, a CpG located in gene *MEST* (Figure s2b)*,* was significantly regulated by a *POE_mQTL_ x Modifier* interaction between its mQTL rs142053217 and ‘predicted mRNA expression levels of *KDM1A*’ (*P_dis_*=1.35 × 10^−6^*, P_rep_*=6.09 × 10^−4^, t-test. *KDM1A* is located in chromosome 1), with lower expression of *KDM1A* increasing the contrast in methylation levels of cg22592140 between heterozygous groups of the mQTL (Table 1, Figure s3b)*.*

### 3.2 Localization of regulatory SNPs contributing to the genetic modification effect

**Figure 6:**
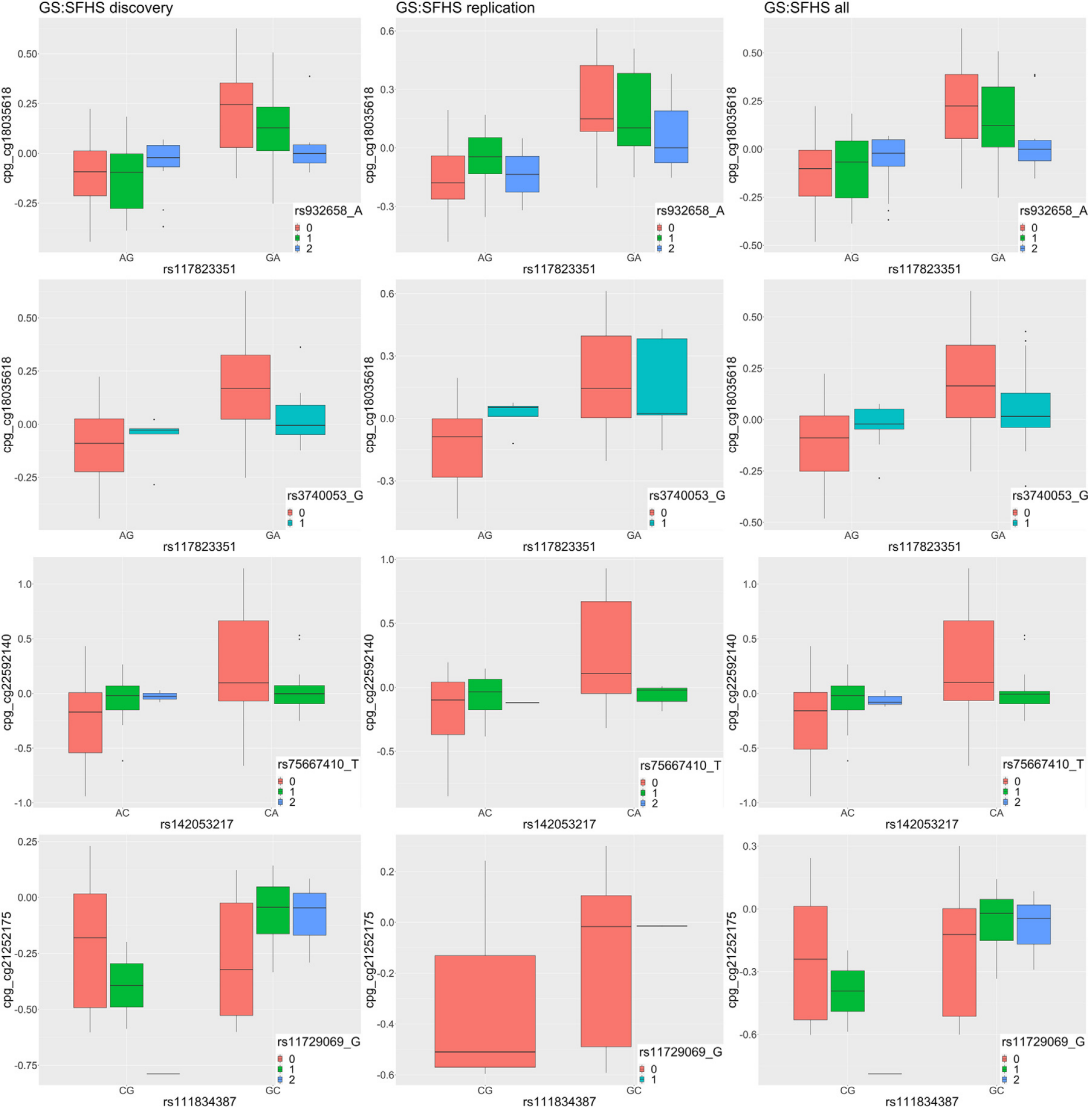
The three modifiable-POE-targeted CpGs were also significantly regulated by *POE_mQTL_ x SNP* interaction effects between the mQTLs and the SNPs used to drive the genetic modifiers. The contrast in methylation levels of the candidate CpGs in mQTL heterozygotes varied depending on the allelic dosage of the SNP used to derive the corresponding genetic modifier. *due to the limitation of sample size, minor homozygous/heterozygous genotype groups were missing in some tests.

Considering that the three identified genetic modifiers (‘predicted mRNA expression levels’ of *SIRT1, TET2, KDM1A*) are essentially weighted combinations of allelic scores at multiple regulatory SNPs, we next tested whether the genetically driven modification effects we detected here can be recapitulated by the *POE_mQTL_ x SNP* interactions between mQTLs and the SNPs used to derive the significant genetic modifiers. Since ‘predicted mRNA expression’ of *SIRT1, KDM1A* and *TET2* was derived from two, one and two SNPs respectively by the MASHR method in PrediXcan (see Methods) [39], we tested *POE_mQTL_ x SNP* interactions for those 5 SNPs (N_test_=5). We identified four of the five SNPs significantly interacting with the POE_mQTL_, that is, regulating the CpGs where the interaction effect was initially detected (Table 2, Figure 6). For example, for cg18035618 we detected a significant interaction effect (*P_dis_*=3.69 × 10^−9^, *P_rep_*=4.33 × 10^−3^, t-test) between rs932658, a SNP used in the prediction model for *SIRT1* expression, and rs117823351, the mQTL that significantly interacted with ‘predicted mRNA expression levels of *SIRT1*’. When accounting for these significant *POE_mQTL_ x SNP* interaction effects as conditional items in the interaction model for the three genetic-modifiers, the interaction effects at the genetic-modifier-level (*POE_mQTL_ x Modifier_Genetic_*) were reduced to a non-significant level, suggesting the leading role of *POE_mQTL_ x SNP* underlying the significant interaction effect from genetic modifiers we detected here (Table s12).

**Table 2:** SNPs that significantly interact with POE (*POE_mQTL_ x SNP*) and the regulated CpGs

mQTL-CpG		SNP modifier		Discovery				Replication		
CpG: id	mQTL: id	SNP id	Relation to G modifier	P(dis, t-test)	Adjust_P(dis,t-test)	Est(dis)	N(dis)	P(rep,t-test)	Est(rep)	N(rep)
cg22592140	rs142053217	rs75667410_T	SNP used in predict *KDM1A* exp	1.35E-06	6.75E-06	0.18	1607	6.09E-04	0.18	645

cg21252175	rs111834387	rs11729069_G	SNP used in predicting *TET2* exp	1.05E-08	5.25E-08	0.18	1607	Not enough data*		
		rs7661349_T		3.51E-02	1.76E-01	-0.06	1607	Not significant in Discovery sample		

cg18035618	rs117823351	rs932658_A	SNP used in predict *SIRT1* exp	3.69E-09	1.85E-08	-0.07	1607	4.33E-03	-0.06	646
		rs3740053_G		1.55E-04	7.75E-04	-1.00E-01	1607	6.01E-03	-0.10	646

dis: discovery sample, rep: replication sample. N: sample size. P(dis, t-test)/P(rep, t-test) and Est(dis)/Est(rep): P values (t-test) and coefficients for the POE_mQTL_ x SNP interaction in the interaction model in discovery/replication samples. Adjust_P(dis, t-test): Bonferroni method adjusted P(dis, t-test). *, due to the relatively small minor allele frequency (MAF) of rs111834387(MAF=0.01) and the limited sample size of replication sample, there was not enough data for this test in replication samples. Number of individuals within heterozygous groups available for testing *POE(rs111834387) x SNP(rs11729069)* interaction effect on cg21252175 in replication samples were shown in Table s11.

### 3.3 Proteins associated with modifiable-POE-regulated methylation sites

Having identified CpGs regulated by the modifiable POE, we next examined if any health-related phenotypic variation can be linked to methylation variations of the CpGs in this class. Plasma protein levels are molecular phenotypes which are closer to the methylation layer as compared to higher layer complex phenotypes such as diseases and behavior traits. Thus, CpG-protein association analysis could provide important insights of functional variation associated with modifiable-POE-regulated CpGs. The ORCADES cohort simultaneously obtained plasma levels of 1092 proteins measured by the Olink Proseek multiplex panel and genome-wide DNA methylation data (N_sample_ =940), which allowed the identification of proteins associated with the CpGs regulated by modifiable POE. To note, the 1092 proteins assayed by the Olink panels applied are already functionally important – they are proteins relevant to diseases and biological process (cardiometabolism, immune response, neurology etc.). The results showed only one CpG-protein pair passed Bonferroni-based multiple testing correction (N_test_=3,276, full results in Table s13): cg21252175 was significantly associated (in *trans*) with plasma protein level of CLEC4C, a protein from the Olink immune-response panel (Beta=0.049, *P_adj_=0.002*, t-test).

Since cg21252175 is located in the UTR3 of gene *ZNF609*, we further examined whether the association between cg21252175 and CLEC4C protein levels implied a link between *ZNF609* and *CLEC4C*. Using data from MESA study [28], a significant and positive correlation was detected between methylation levels of cg2125217 and mRNA levels of *ZNF609* in both CD4+ peripheral T cells (R=0.15, *P*=0.03, Spearman) and CD14+ peripheral monocytes (R=0.15, *P*=1.52 × 10^−6^, Spearman, Figure s4a). Using whole blood data from the GTEx consortium through the GEPIA portal [29,40], mRNA expression level of *ZNF609* was significantly correlated with mRNA expression levels of *CLEC4C* at population level (R=0.21, *P*=1.1 × 10^−4^, Spearman, Figure s4b). Using a single-cell RNA-seq data of PBMC in an adult donor, mRNA expression levels of *ZNF609* and *CLEC4C* were significantly correlated at individual level (R=0.36, *P*=0.0002, Spearman, Figure s4c).

### 3.4 Phenotypes associated with modifiable-POE-regulated methylation sites

**Figure 7:**
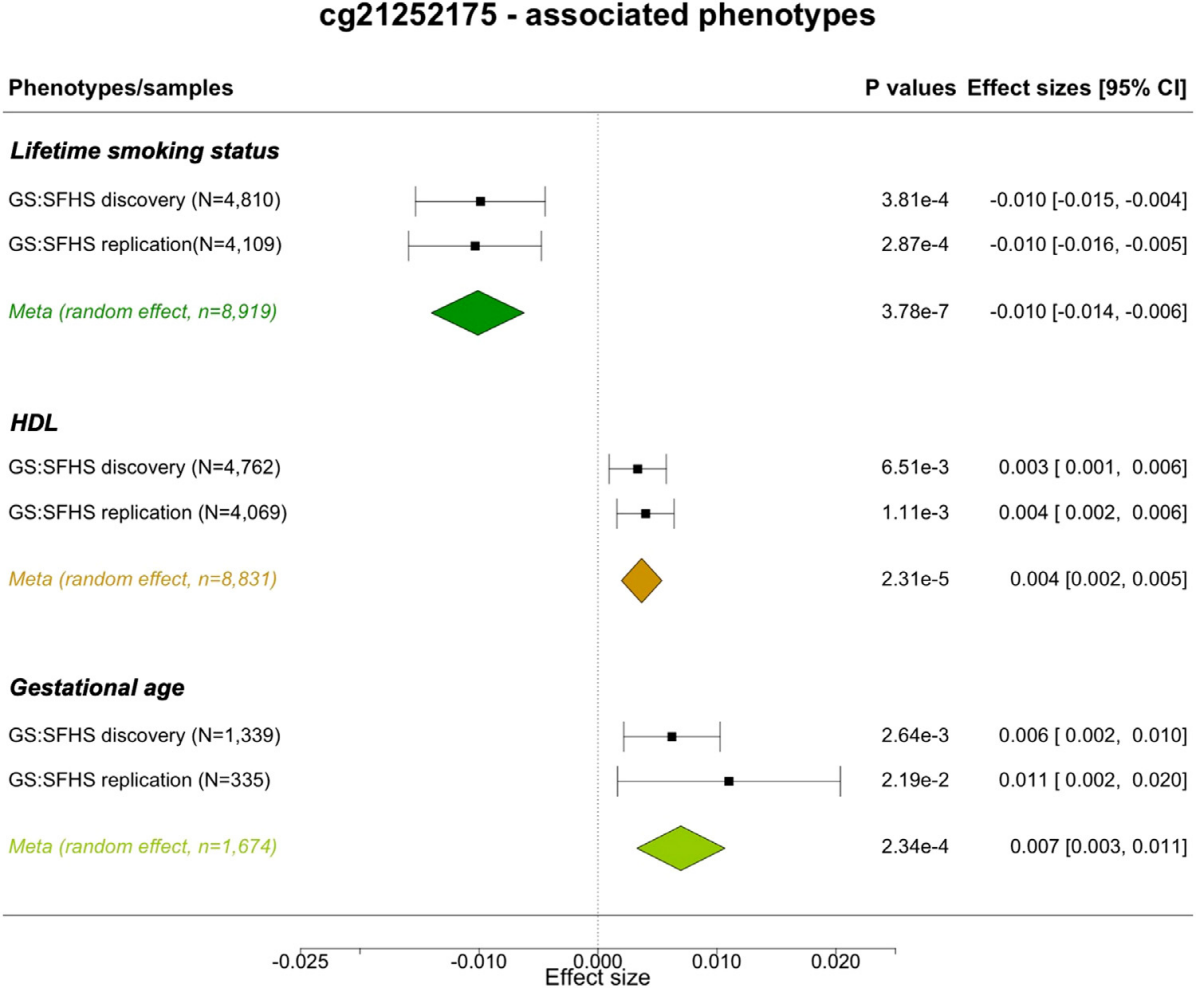
Forest plot for phenotypes associated with cg21252175. HDL: high-density lipoprotein. Meta: Meta-analysis performed using the random effect model.

To explore the association between variation in CpGs targeted by modifiable POE and health/disease-related phenotypes, we collected 79 phenotypes in GS:SFHS (Table s7). Phenome-wide association tests relating methylation levels to phenotypes were performed for the three identified modifiable-POE-regulated CpGs using the whole GS:SFHS methylation sample (meta-analyzed across discovery (N_sample_=5081) and replication (N_sample_=4445) samples; total N_sample_=9526.). After Bonferroni-based multiple testing correction (N_test_=79 × 3=237), two CpG-phenotype associations reached phenome-wide significance: cg21252175 was both associated with ‘lifetime smoking status’ (*P_adj_*= 9.0 × 10^−5^, t-test) and high-density lipoprotein (HDL) levels (*P_adj_* =0.006, t-test) (Figure 7, Table s8). Although limited by sample size (Figure 7), cg21252175 was also associated with gestational age (measured as weeks at birth) at an adjusted P ≤ 0.06 level (*P_adj_*=0.056, t-test). These associations displayed consistent patterns across discovery and replication samples (Figure 7, Table s8).

## 4 Discussion

In this study, we reported significant and replicated modification effects from both genetic and environmental variables on the parent-of-origin effect that affects DNA methylation levels at three CpGs. Identified environmental modifiers included ‘lifetime smoking status’; genetic modifiers included ‘predicted mRNA expression levels’ of several DNA methylation/imprinting machinery genes (*SIRT1, TET2, KDM1A)*. Importantly, we found that both genetic and environmental modifiers were targeting the same CpG (cg18035618). These provided evidence for a special type of CpGs in the human genome regulated by parent-of-origin-effects that are modulated by genetic or environmental modifiers. We further found that these CpGs are likely to be phenotypically relevant: for molecular phenotypes, DNA methylation level at one the CpG, cg21252175, was associated with protein levels of the immune-response-related protein CLEC4C. For non-molecular phenotypes, this CpG was associated with ‘lifetime smoking status’, HDL levels and gestational age(the latter at the suggestive significance level).

Statistically, the model we proposed here for detecting modifiable POE is built on a previous POE-mQTL model which we used to localize regulatory mQTLs for the POE-influenced CpGs (model 1 in Methods) [4,24]. For those CpGs, one of the hallmarks of the POE-regulation was the methylation difference between the two heterozygous mQTL genotype groups (Figure 1) [4,6]. Here, our new interaction model tests whether that difference remains stable or varies under certain conditions, that is, if the parent-of-origin effects are the same or different across different environments or in different genetic backgrounds. Biologically, this implies the existence of a new and different layer of regulation for DNA methylation: genetic/environmental factors could influence the level of DNA methylation of CpGs, not only through direct effects, but also through interacting with the POE (Figure 1).

Our results support this hypothesis. Smoking has been widely studied for its direct influence on DNA methylation [41,42] and its interactions with additive genetic effects on methylation levels [43]. Here, for the first time, our study reported that smoking could also affect DNA methylation variation indirectly through interaction with a non-additive genetic effect, POE. Similarly, variation in DNA methylation and imprinting machinery genes, either in the forms of variable expression or mutation, have been known to directly affect DNA methylation. SIRT1 regulates DNA methylation and protects methylation at imprinted loci by directly regulating acetylation of DNMT3L, which is required for the establishment of maternal genomic imprints [12,31]. TET2 promotes DNA demethylation by converting 5-methyl-cytosine to 5-hydroxymethyl-cytosine and is required for demethylation at imprinted loci in the germline [12,32]. KDM1A removes methylation of histone H3K4 in imprinted genes*,* its deficiency is associated with alterations in DNA methylation and expression at imprinted genes [12,33]. Here, our results provided human-study evidence in vivo for the role of these machinery genes in POE-related methylation variation. Importantly, for the first time we showed that besides direct effects, these genetic factors could introduce indirect regulatory effects on DNA methylation levels through interactions with POE.

The detection of interactions between genetic modifiers and POE led us to further identify significant and replicated *POE_mQTL_ x SNP* interaction effects between mQTLs and SNPs used in imputing genetic modifiers. One important feature of the genetic modifier variables we derived here is that they represent the proportion of mRNA variation determined by germline genetic variation, which is constant and stable throughout the life [39]. Our results demonstrated that an individual's genetic background of DNA methylation and imprinting machineries has the potential to modify POE. The localization of the genetic-based modification effect at regulatory SNPs of these DNA methylation and imprinting machinery genes strongly supports this, and importantly, indicated that genetic variation in machinery genes is an important source of epistasis. One of the machinery genes, *SIRT1*, has been well known for its role in mental health disorders such as depression [44], but very few studies have examined its role as a modifier for non-additive genetic effect such as POE. Our result revealed a new potential path of variation in this gene to introduce molecular differences.

The reason why POE can be modified is deeply rooted in the unique nature of genomic imprinting: established at an early developmental stage, needing to be protected from global-demethylation and maintained throughout the lifespan [12]. These complex and multi-stage processes have been shown to be vulnerable to environmental fluctuations and involve delicate regulation of multiple gametic and zygotic genetic factors, including *TET2, SIRT1, KDM1A* as we identified here [11,12]. Indeed, the vulnerability of at least a subset of POE-CpGs was revealed here, as the *POE_mQTL_ x Modifier* interaction effect explained a non-negligible proportion of methylation variance (1%-2.2%), and that at least one (cg18035618) of the three CpGs identified was targeted by independent environmental and genetic modifiers. The detection of modifiable POE on cg22592140, a CpG located in *MEST* was in line with a previous study suggesting the methylation status in the *MEST* region is vulnerable to perturbations as compared to other imprinted regions, likely due to its later methylation acquisition kinetics [45]. The third modifiable-POE influenced CpG, cg21252175, was associated with CLEC4C, an immune-response transmembrane protein treated as a marker gene for plasmacytoid dendritic cells [46]. This CpG was also associated with ‘lifetime smoking status’, HDL levels and gestational age (the latter at the suggestive significance level) (Figure 7) in our analysis, and was previously found to be associated with maternal body mass index/overweight/obesity [47]. These convergent lines indicated that this newly uncovered class of vulnerable POE-CpGs may play an important role in connecting early life stress, variations in genetic background and later life health issues.

The three modifiable-POE regulated CpGs are likely to be important for the POE patterns in local regions. Two of the three CpGs were located in ICR, the third CpG was located within CTCF binding site(Figure s1). ICR are crucial control regions usually found to be responsible for the POE patterns of large ranges of nearby regions [48], regional regulatory mechanisms through ICRs have been constantly linked to the binding of CTCF [12], and CTCF binding is influenced by DNA methylation [49]. These facts, combined with that all three CpGs display active epigenetic activity (overlap with H3k4me1, H3k4me3, H3k27ac narrow peaks), may implicate the potential of regional influences from the modification of POE through regulating modifiable CpGs in ICR regions.

Beyond the complexity of POE-regulation revealed here, our observations that both a person's genetic and environmental background can modify how a POE-mQTL regulates its target CpG demonstrates an important general principle for medical genetic research and relevant clinical applications which is that it can be important to account for the environmental context and the genetic background. DNA methylation has been proposed as promising regulatory target for pharmaceutical interventions [50], but for any clinical application relating to CpGs subjected to modification effects, it would be necessary to consider both genotype and environmental exposures when tailoring any intervention. Taking cg18035618, its mQTL rs117020124 and the modifier “lifetime smoking status” as an example, if we do not account for lifetime smoking status, carriers of the AG genotype (that should be distinguished from the GA genotype) of the mQTL rs117020124 displayed lower methylation levels for cg18035618 when compared to non-carriers; when accounting for lifetime smoking status, we found AG genotypes carriers could be further stratified and that carriers who are also current smokers display the lowest methylation level of the CpG (Figure 5a).

There are limitations in this study. First, mRNA expression levels used here were predicted and only reflect genetically influenced expression variation (and not necessarily all of it). Future studies examining measured mRNA expression will be necessary to account for the modification effect from the environmentally determined fraction of mRNA expression. Second, the analyses performed here simultaneously require DNA methylation data and SNP data with information of parent-of-origin of the alleles transmitted to the offspring. Our sample size was relatively limited in size, only focuses on CpGs which previously were found to show parent of origin effects and is only based on DNA from one tissue (blood) and in individuals who were relatively young (mean age = 34). Thus we may well underestimate the scale of interactions with environmental effects or the genetic background that affect DNA methylation. Further work would benefit from larger sample sizes from multiple tissues and across a wide range of ages as well as further validation in other cohorts. Finally, the exact time window or developmental stage in which each environmental modifier exerted their influences remains unknown. Cohort data with higher resolution of environmental exposure records, in particular those measuring early life exposures, will be crucial to understand the vulnerable stage or stages for CpGs influenced by modifiable POE.

In conclusion, we provided population-level evidence for modification effects from multiple genetic and environmental factors on parent-of-origin-effects at the DNA methylation level. A subset of parent-of-origin-effect-influenced CpGs that are vulnerable to modification effects were uncovered, which opens new questions for future profiling of the modification patterns and phenotypic consequences of this class of CpGs.

## Contributors

YZ, CSH, and PN conceived and designed the analyses presented in this manuscript, and supervised the study. DJP, AMM, CSH, KLE and JFW contributed to conceive and design the study populations and phenotypic recording, and funding acquisition. YZ, CG and SH conducted the analyses. CA, RW, SWM, AC, YZ, CH, AF, RAM and MJA managed and maintained the data and performed quality control and data annotation. YZ, CSH, CA, and PN wrote the paper (original draft). All authors reviewed and edited the paper. YZ and CG directly accessed and verified the underlying data. All authors discussed results, read, and approved the final manuscript.

## Data sharing statements

Summary statistics supporting the conclusions of this article are included within the article and its additional files. GS:SFHS: Generation Scotland data are available from the MRC IGC Institutional Data Access / Ethics Committee for researchers who meet the criteria for access to confidential data. Generation Scotland data are available to researchers on application to the Generation Scotland Access Committee (access@generationscotland.org). The managed access process ensures that approval is granted only to research which comes under the terms of participant consent which does not allow making participant information publicly available. ORCARDES: there is neither research ethics committee approval, nor consent from individual participants, to permit open release of the individual level research data underlying this study. Please contact the QTL Data Access Committee (accessQTL@ed.ac.uk) for further information if required.

## Declaration of Competing Interest

Dr. McIntosh reports grants from Janssen, grants from The Sackler Trust, personal fees from Illumina, personal fees from Janssen, during the conduct of the study; Dr. Haley reports grants from Medical Research Council, grants from Wellcome Trust, grants from Chief Scientist Office of the Scottish Government Health Directorates, grants from Scottish Funding Council, during the conduct of the study; Dr. Navarro reports grants from Medical Research Council, grants from Wellcome Trust, grants from Chief Scientist Office of the Scottish Government Health Directorates, grants from Scottish Funding Council, during the conduct of the study. Dr. Bretherick reports grants from The Wellcome Trust, during the conduct of the study.

## Appendix Supplementary materials

Image, application 1

Image, application 2

Image, application 3
