# Supplementary material for: Lifestyle and Genetic Factors Modify Parent-of-Origin Effects on the Human Methylome
Source: eBioMedicine. 2021 Dec 6;74:103730. doi: 10.1016/j.ebiom.2021.103730 (PMC8654798; doi:10.1016/j.ebiom.2021.103730)
Supplement: Supplementary file 3 [file mmc3.docx]

supple_materials_12-11-2021.docx: Figure s1-4, Text s1-4.

supple_tables_12-11-2021.xlsx: Table s1-13.
